# Determinants of variability in motor performance in middle childhood: a cross-sectional study of balance and motor co-ordination skills

**DOI:** 10.1186/2050-7283-1-29

**Published:** 2013-12-17

**Authors:** Patricia K Kitsao-Wekulo, Penny A Holding, Hudson Gerry Taylor, Jane D Kvalsvig, Kevin J Connolly

**Affiliations:** KEMRI/Wellcome Trust Research Programme, Centre for Geographic Medicine Research –Coast, Kilifi, Kenya; International Centre for Behavioural Studies, Nairobi, Kenya; Case Western Reserve University, Cleveland, OH USA; University of KwaZulu-Natal, Durban, South Africa; Department of Psychology, The University of Sheffield, Sheffield, UK

**Keywords:** Motor performance, Resource-constrained setting, Rural, School-age, Variability

## Abstract

**Background:**

Physical activity is a key component of exploration and development. Poor motor proficiency, by limiting participation in physical and social activities, can therefore contribute to poor psychological and social development. The current study examined the correlates of motor performance in a setting where no locally validated measures of motor skills previously existed. The development of an appropriate assessment schedule is important to avoid the potential misclassification of children’s motor performance.

**Methods:**

A cross-sectional study was conducted among a predominantly rural population. Boys (N = 148) and girls (N = 160) aged between 8 and 11 years were randomly selected from five schools within Kilifi District in Kenya. Four tests of static and dynamic balance and four tests of motor coordination and manual dexterity were developed through a 4-step systematic adaptation procedure. Independent samples t-tests, correlational, univariate and regression analyses were applied to examine associations between background variables and motor scores.

**Results:**

The battery of tests demonstrated acceptable reliability and validity. Variability in motor performance was significantly associated with a number of background characteristics measured at the child, (gender, nutritional status and school exposure) household (household resources) and neighbourhood levels (area of residence). The strongest effect sizes were related to nutritional status and school exposure.

**Conclusions:**

The current study provides preliminary evidence of motor performance from a typically developing rural population within an age range that has not been previously studied. As well as being culturally appropriate, the developed tests were reliable, valid and sensitive to biological and environmental correlates. Further, the use of composite scores seems to strengthen the magnitude of differences seen among groups.

## Background

The processes that take place in gross and fine motor development allow children to explore the spatial properties of their environment and the functional properties of the objects in it. This exploration in turn facilitates general development and supports the achievement of healthy and independent functioning in everyday life. Poor motor proficiency, therefore, interferes with participation in physical and social activities and is likely to be associated with limitations in multiple spheres of development (Skinner and Piek 2001).

As with many areas of development, motor skills follow a sequential and predictable pattern (Berk 2006) that is comparable among children. However, differences in environmental context and in parenting strategies lead to observable precocity in African infants in early motor development (Leiderman et al. 1973). Little is known about the later influences upon variability in motor performance amongst a normal population of school-age children in the African setting. Attempts to develop culturally valid measures of psychomotor development or to establish normative standards for African children (Abubakar et al. 2008a; Gladstone et al. 2010) have focussed primarily on infants and preschoolers. The consequent lack of locally validated measures of motor development for school-age children may limit the reliability of measurement and lead to mis-classification of children (van de Vijver and Tanzer 2004; Connolly and Grantham-McGregor 1993). Given the widely reported precocity of motor development among African children (Warren 1972; Super 1976), existing norms for measures published in western settings may therefore not be appropriate. In addition, in the rural East African context and in similar settings, assessment protocols need to address the lack of available staff with previous assessment experience, limited resources for purchasing expensive published tests and equipment, and the issue of engaging children who are unused to standardized testing procedures.

Bronfenbrenner’s bioecological model (Bronfenbrenner and Ceci 1994) posits that a child’s development is determined by both proximal and more distal influences. The rate of motor progress of healthy children is therefore susceptible to the influence of several interrelated factors and contributes to variability in motor skill proficiency (Lotz et al. 2005). These include internal (biological) factors such as gender and age (Largo et al. 2003). Other background characteristics may impact on motor development through their influence on experience, and or by altering brain development and function (Walker et al. 2011). Previous studies in Africa and other low resource settings have indicated multiple influences upon variability in motor proficiency including nutritional status (Wachs 1995; Stoltzfus et al. 2001), HIV, malaria and helminthic infections e.g. (Olney et al. 2009; Botha and Pienaar 2008; Bagenda et al. 2006), poverty, poor health and unhealthy environments (Grantham-McGregor et al. 2007; Evans 2006), and the lack of opportunities for play (Gallahue and Ozmun 2002).

To reliably identify deviations from normal progress, it is necessary to have tools that have been validated in context. The measurement of motor proficiency in the current study was part of a larger study that focused upon developing a methodology to examine the longer-term effects of central nervous system (CNS) infections (such as malaria, meningitis and neonatal sepsis) endemic to the region. Previous studies have suggested that while the effects of these infections in the brain may be diffuse (Holding and Boivin 2013), in the longer-term, larger effect sizes are commonly seen in more complex tasks associated with executive functions. The primary objective of this study was therefore to describe the motor performance of a sample of school-age children from coastal Kenya through the examination of associations of motor performance with socio-demographic factors. To achieve this objective, a battery of motor assessments was developed that would be reliable, valid and sensitive to the long-term developmental consequences of health-related risk factors in our target population.

## Methods

### Design

This cross-sectional study was undertaken as part of a programme to develop appropriate methodology for the neuropsychological assessment of school-age children in coastal Kenya. The larger programme included children aged between 8 and 11 years, covering the stage of development where it becomes easier to measure discrete areas of performance.

### Study setting

The study was conducted at the Kenya Medical Research Institute’s Centre for Geographic Medicine Research in Kilifi District at the Kenyan Coast. The area covered is a predominantly rural community mainly engaged in agriculture with few and unstable income-generating opportunities (FAO Kenya 2007). More than half the population lives in absolute poverty, surviving on less than USD 2 per day, with high illiteracy levels increasing the population’s vulnerability to food insecurity and to endemic tropical infections (Kahuthu et al. 2005; Kenya National Bureau of Statistics (KNBS) and ICF Macro 2010). At the time of the study, the district had 230 primary schools with a total enrolment of 137,958 (75,582 males and 62,376 females) children. Primary school enrolment rates within the district were low at 66.5% (Kahuthu et al. 2005).

A typical home in Kilifi comprises a large homestead with several small huts in which extended family members live together and share in the daily household chores. It is not uncommon for members from different generations to share in child-rearing duties. Children of school-going age spend a lot of their time outdoors. Boys have a more unstructured time, engaging in mostly play activities, while girls attend to chores such as fetching firewood and water and helping their mothers in the fields (Wenger 1989).

### Sampling and sample characteristics

School-age children were selected through stratified sampling from the catchment area of five randomly selected local schools distributed across neighbourhoods ranging from sparsely populated to semi-urban areas (Kitsao-Wekulo et al. 2012). Both school-going and non-school going children were identified for inclusion. At the time of the study, the selected schools had a total population of 2,755 children. A total of 308 children were recruited to represent the diverse geographical areas, represented by equal numbers of boys and girls, in each of three age bands – 8, 9 and 10 years. Additional child level characteristics included length of school experience and nutritional status (defined by the presence or absence of growth retardation). Birth records were used where available to confirm age. In cases where records were not available, the child’s age was estimated by using major local or national events that occurred around the time of the child’s birth. School exposure was defined as each year of enrolment from nursery class. Household-level characteristics comprised an index of household resources that divided the sample into three approximately equal groups from the least wealthy to the most wealthy (Level 1, Level 2 and Level 3).

### Ethical considerations

The Kenya Medical Research Institute/National Ethics Review Committee (KEMRI/NERC) provided ethical clearance for the study. Permission to visit schools was obtained from the District Education Office. We explained the purpose of the study to the head teachers of selected schools and then sought their permission to recruit children. We also held meetings with community leaders, elders and parents (or guardians) of selected pupils to explain the purpose of the study. After each meeting, a screening questionnaire was administered to establish if selected children met the study’s eligibility criteria. We presented information on the study to parents in the language with which they were most familiar. We then obtained written informed consent for their children’s participation. All the selected children assented to their participation in the study.

The Ten Questions Questionnaire TQQ (Mung’ala-Odera et al. 2004) and observation by the assessment team were used to establish any visual, auditory and motor impairment, as well as other serious health problems in children. Children who were found to be physically unable to perform the tests, due to severe limitations in physical and global mental functioning, were excluded.

### Development of motor tests

In the development of the battery, we followed the 4-step systematic test adaptation procedure outlined by Holding, Abubakar and Kitsao-Wekulo (2009).

#### *Step 1: Construct definition*

The focus of the battery was tasks that measured balance and co-ordination, as these skills reflect planning of movements that may be more reflective of an underlying executive function component of motor proficiency. We therefore defined motor proficiency as the specific abilities measured by tests of balance, bilateral co-ordination, upper limb co-ordination, visual-motor control and upper limb speed and dexterity (Sherrill 1993).

#### *Step 2: Item pool creation*

Some tests were modelled after those in the Movement – Assessment Battery for Children (Movement-ABC; Henderson and Sugden 1992), a battery of motor tasks designed for children ages 5–12 years. Apart from the fact that it takes a short time to administer, the most important advantages of the Movement-ABC compared with other available tests are its cross-culturally applicability, simplicity of instruction and demonstration and the ease with which trainers can be trained in administration (Cools et al. 2009). Additional tests in the battery, such as the Bolt Board Test, were conceptualised and designed by the investigation team.

#### *Step 3: Developing the procedure*

We produced a manual of instructions for the newly created tests and modified existing items and procedures to suit the cultural norms and practices of the study context. Instructions were formulated in the local language. Tasks were chosen on the basis that their requirements were familiar to children and that they were similar to activities that children regularly engaged in. The appropriateness of the procedures was pilot-tested on groups of between 10 and 20 children. Some of the instructions were rewritten to improve clarity.

We initially piloted the following tests: fine motor tests including the Bolt Board, Pegboard and Bead Threading Tests; tests of dynamic balance included Hopping in Squares, Jumping in Squares (with two feet together), Jumping and Clapping, and the Ball Balance Tests; Static balance tests included Standing on One Leg, One Board Balance and Two Board Balance Tests. We established the ceiling and floor effects on each test. Very easy items on which 30% or more of the children made no errors like Jumping in Squares were dropped. Very difficult items on which 20% or more of the children were unable to reach the first level (e.g. for some children with wide feet, the requirement to balance on two ridged boards on the Two Board Balance Test was impossible to achieve) were dropped. The Standing on One Leg Test, in which one leg was held off the ground, was modified as the Stork Balance Test as assessors were not able to establish the angle at which the free leg was held, especially for girls wearing long skirts.

The process of pilot testing continued until there was no further need for modifications and children were deemed to have understood the test requirements. In this manner, the number of modifications made determined the total number of children on which the tests were pilot-tested, as additional children were included as needed. Four assessors with professional backgrounds in education (varying from diploma to degree level) were trained in administration and scoring of the gross and fine motor tests. Training included participation in the initial development of instructions for test administration and selection of the tests, as well as direct instruction and practice in administration procedures.

#### *Step 4: Evaluation of modified tests*

Once the content and format of the assessment tasks were established, extensive practice sessions in which assessors administered tests to 30 non-study children under the close supervision of the PI, enhanced standardisation in the administration procedure. These non-study children were divided into three groups of 10 each comprising 5 younger (7–8 years) and 5 older (10–11 years) school-going and non-schooling children. Each group was administered a set of tests within the three categories – fine motor and tests of static and dynamic balance.

The final battery of motor tests comprised 8 tests, five tests of gross motor abilities covering static and dynamic balance – and three timed tests of manual dexterity to assess eye-hand coordination.

### Data collection procedures

#### *Background characteristics*

We measured children’s heights using a stadiometer. The child was asked to remove his/her shoes, place the feet together and stand with his/her back and head against the board. The child was instructed to stand up straight and look straight ahead. The moveable headpiece was then brought onto the uppermost point of the head with sufficient pressure to compress the hair. One assessor was designated to take the reading, while another noted it down on a paper. Two readings were taken for each child. The measurement was recorded to the nearest 0.1 cm. Growth retardation was defined as height that was more than 2 standard deviations below levels predicted for age according to the World Health Organization reference curves for school-aged children (World Health Organization 2007). School exposure was measured as the number of complete years that the child had attended school.

The constituent items of the wealth index score were developed through a review of indicators of socioeconomic status (SES) made in the study population, as well as a local investigation of household characteristics associated with educational outcome (Holding & Katana, internal report). It was calculated by summing the values assigned to each of six SES variables obtained through parental interview: parental education and occupation (mothers and fathers separately), ownership of small livestock and types of windows in the child’s dwelling place. Education groupings were calculated on the basis that primary education takes 8 years to complete, post-primary education takes between 9 and 12 years to complete while a tertiary education certificate is obtained after more than 12 years of education, thus: ‘0’ = no education; ‘1’ = <8 years of education; ‘2’ = 8 years of education; ‘3’ = 9–12 years of education; and, ‘4’ = >12 years of education. Parental occupation was denoted thus: ‘0’ = not known/deceased; ‘1’ = unemployed/housewife; ‘2’ = subsistence farmer; ‘3’ = unskilled/petty trader; ‘4’ = semi-skilled; and, ‘5’ = skilled. The number of livestock was coded as ‘0’ = none, ‘1’ = <5, and ‘2’ = 5+ while the type of windows was coded ‘0’ = none, ‘1’ = open, ‘2’ = small, ‘3’ = wooden, ‘4’ = wire, and ‘5’ = glass.

#### *Test administration*

The motor tests were administered to 148 boys and 160 girls (N = 308) aged between 8 and 10 years as part of a neuropsychological battery. The full battery consisted of the following tests: a non-verbal Tower test of problem-solving and planning ability; the Self-Ordered Pointing Test to assess verbal-visual selective reminding; Verbal List Learning to test learning and working memory; a non-verbal test of memory (Dots); a Contingency Naming Test of executive function to assess response inhibition, attentional shift and cognitive flexibility; a Score test of auditory sustained and selective attention; the People Search test of visual sustained and selective attention; and, the Coloured Progressive Matrices which assessed non-verbal reasoning. These tests are described in detail by Kitsao-Wekulo and colleagues (2012).

Lateral preference (hand and foot) was assessed to establish on which side testing should begin, as all tests required the assessor to begin with the preferred limb. We asked the child to demonstrate a variety of lateralized tasks with the hand (show me how you throw an object) and foot (show me how you kick a ball) (Denckla 1985). The tests were administered outside in an open flat area away from other children to avoid distractions. Each child was tested individually but within sight of other children, and in familiar surroundings to minimise test anxiety. To improve standardisation in administration, care was taken to ensure that the testing environment in all the schools was as similar as possible. Most children were able to complete the motor tests in 30 minutes, with times ranging from 23 to 46 minutes. Assessors who were native to the study area and who were fluent in both testing languages provided instructions in the language with which children were most familiar.

##### Stork Balance Test

This was a test of static balance. The test was administered by asking the child to stand on one leg with the hands on the hips. The second non-standing foot rests on the knee. The child completed the task first on the preferred leg, then on the non-preferred leg with the eyes open and eyes closed. A second trial on each leg was administered if any errors were made within 30 seconds of the first trial. Errors included placing the non-standing foot on the ground and removing the hands from the hips. The trial with the highest time was noted. Percentile cut-offs for the entire sample were calculated and scores ranging from ‘0’ (complete failure) to ‘3’ (complete pass) were awarded based on the highest time achieved. To provide a continuous score, the scores across the four conditions were summed.

##### Ball Balance Test

In this test of dynamic balance, the child was asked to walk along the outline of the perimeter of a rectangle marked with a rope placed on the ground. This task was completed while balancing a tennis ball on a square board using an outstretched arm. On the first trial, if the ball dropped up to 10 times, or if the child made any of the following errors (does not resume walking from the point of drop, supports the ball with the free hand or places the thumb on the upper surface of the board), a second trial was administered. If the ball was dropped up to 10 times again on the second trial, a third trial was administered with the arm bent. The child’s score was calculated according to the number of ball drops on each trial.

##### Hopping in Squares Test

This test in which the child hopped in five squares marked on the ground with a rope was a test of dynamic balance. The task was completed first on the preferred leg then on the non-preferred leg. Errors were recorded if the child stepped onto the rope, made two hops in one square or hopped outside the square. An acceptable landing was defined as coming down on one foot with the sole of the foot meeting the ground within the last square. If the child was successful on the first trial, a score of ‘2’ was awarded for each of the three aspects (no errors, five correct hops and acceptable landing) and for each leg separately. If the first trial was not completed accurately, a second trial was administered. Each of the three aspects was scored ‘1’ if success was achieved on the second trial. The child scored ‘0’ if s/he did not achieve success on all three aspects. The total score was calculated by summing the scores for errors, hops and landing for both legs.

##### Jumping and Clapping Test

This test was administered to assess dynamic balance. The child was asked to jump as high up in the air as possible and to clap the hands while the feet were in the air. The number of claps for each of three trials was recorded. The child’s score was the highest number of correct claps.

##### One Board Balance Test

In another test of static balance, the child was asked to balance on a ridged board, first with the preferred leg (then with the non-preferred leg) on the board and the other in the air while being timed. A second trial was administered if any errors occurred within a 30-second time period. As with the Stork Balance Test, percentile cut-offs based on the highest time achieved on each leg were calculated. Scores ranging from ‘0’ (complete failure) to ‘3’ (complete pass) were awarded and summed to derive a continuous total test score.

For the timed fine motor tests, the assessor first demonstrated the correct procedure for completion and then allowed the child a practice trial. When the child demonstrated that they had understood the task requirements, the assessor gave the instruction ‘Do this test as quickly as you can without making any mistakes’ and then began to time the test.

##### Bolt Board Test

This was a test of manual dexterity. The child was presented with a board of nuts on which were screwed 20 bolts in four rows of five. There were red-coloured bolts on two rows on one side and blue-coloured ones on the other. Beginning with the preferred hand, the child was required to unscrew a bolt from the same side, turn it upside down and screw it back on to the nut. The same process was followed using the non-preferred hand with the bolts on the other side. Alternating between the right and left hand, the bolts were unscrewed and screwed until all 10 on each side had been turned over. Three 60-second trials were administered. The number of bolts completed across the three trials was recorded. The child’s score was derived from the total number of bolts manipulated correctly.

##### Bead Threading

In a second test of manual dexterity, the child was required to thread as many beads as possible onto a shoe lace within 30 seconds. The child’s score was the mean number of beads threaded across three trials.

##### Pegboard Test

The third test of manual dexterity required the child to insert as many pegs as possible into the holes of a pegboard within 25 seconds. This test was completed first with the preferred hand, then with the non-preferred hand and finally with both hands together. Three trials were administered and an average score was calculated for each condition. The child’s overall score was the mean number of pegs across the three conditions.

A second test administration was completed about 6 weeks after the initial administration. To reduce the burden on each child we only administered half of the full battery at re-test. Thus only 149 children were included in the sample to calculate reliability estimates of the motor tests. Five children were not re-tested for various reasons such as relocation from the study area, travelling outside the study area and refusal for continued participation.

### Analysis

The intraclass correlation coefficient (ICC) was used to evaluate test-retest reliability (Portney and Watkins 2000). A paired-samples t-test was conducted to determine whether a practice or learning effect existed between test and retest scores. Age effects were significant for most measures, documenting significant increases in scores with increasing age. Constituent motor tests were therefore age standardized by regressing scores on age. Age-corrected scores were obtained by computing differences between observed and predicted scores in units of standard error of the estimate (i.e., in z-score units).

To discount the influence of outliers, extreme scores below -3 or above 3 were winsorized by replacing their values with the nearest scores within this range. Tests of skewedness and kurtosis confirmed normalcy of score distributions. Maximum likelihood factor analysis with oblique rotation was then applied to the z-scores to reduce the multiple motor scores to ability composites (Ackerman and Cianciolo 2000). Factor analysis yielded support for a two-factor solution; there were few cross-loadings and more than three tests loaded on each factor, with all tests loading above .30 on each. Tests loading on the Motor Co-ordination factor were Pegboard, Bead Threading, Bolt Board and Jumping and Clapping, and those loading on Static and Dynamic Balance were Stork Balance, One Board Balance, Ball Balance and Hopping in Squares (Table 1). Factor scores were defined as the mean of the z-scores for the tests loading on each factor. An Overall Motor Index was also defined as the mean of the two factor scores. A similar procedure was applied on the z-scores of the tests of cognitive functioning to produce factor composites labelled Executive Function and Verbal Memory.

**Table 1:** **Factor loadings of constituent motor tests** ^**a**^

Test items	Factor 1	Factor 2
Pegboard	**.812**	.020
Bead Threading	**.797**	-.077
Bolt Board	**.538**	.025
Jumping & Clapping	**.304**	.120
One board Balance	-.090	**.658**
Stork Balance	.013	**.641**
Hopping in Squares	.168	**.398**
Ball Balance	.063	**.327**

^a^Numbers in boldface are for factor loadings greater than .3.

The standardized scores of these summary variables were used in subsequent analyses. We used Pearson’s correlation coefficient to measure associations of composite motor scores with executive function and verbal memory scores in order to establish convergent and discriminant validity. Independent sample t-tests were applied to examine the effect of gender, nutritional status and area of residence on test scores. Univariate analysis was used to make group comparisons among categories based on school exposure and household resources. Regression analysis was conducted to determine the relative contribution of each background characteristic to constituent tests, factor composites and the Overall Motor Index. For all analyses, *p* < .05 was used to determine statistical significance.

## Results

### Descriptive statistics

The mean age for boys was 9.06 years (SD = 1.05) and 9.10 years (SD = 1.18) for girls. Overall, the mean age for the sample was 9.08 years (SD = 1.16). Noteworthy is the strong ceiling effect seen on the Hopping in Squares Test as nearly half of the sample (compared to between two and twenty percent on the other four tests) obtained the maximum possible score on this test. Nearly 20% of the sample scored ‘0’ on the One Board Balance compared to between two and nine percent on the other tests (Table 2).

**Table 2:** **Distribution of scores and test-retest reliability indices on motor tests**

Tests	Range	% with max score	Mean (SD)		ICC
			Time 1^a^	Time 2^b^	
Stork Balance	0-12	2.9	6.64 (3.30)	4.79 (1.79)	.682
Ball Balance	0-12	20.1	9.17 (2.46)	9.60 (1.93)	.507
Hopping in Squares	0-12	42.1	8.91 (3.51)	10.19 (2.81)	.522
One board Balance	0-6	15.3	2.44 (2.04)	2.81 (2.06)	.511
Jumping and Clapping	0-4	1.6	1.81 (.626)	1.86 (.626)	.730
Bolt Board^c^	2.50-20.50	-	9.07 (2.49)	10.43 (2.74)	.813
Bead Threading^c, d^	3.33-15.33	-	9.73 (1.70)	-	-
Pegboard^c^	3.56-13.56	-	8.68 (1.61)	9.03 (1.77)	.896

^a^n = 308.

^b^n = 149.

^c^No maximum scores as these were timed tests.

^d^No retest data available.

Data were incomplete for 16 children due to limb deformities, inability to maintain balance for at least one second, illness on the day of testing and missed appointments. We assigned scores as follows for these missing data: a score of ‘0’ was assigned if the child was unable to meet basic task demands; if a test was not administered to the child because of an error on the assessor’s part, we assigned the modal score attained on the specific test for a given age-group. Because findings were highly similar when these data were excluded we present results only with assigned scores included.

The following results are presented in Table 2. Test-retest reliability levels ranged from .5 to .9 for seven tests; one test, Bead Threading, was administered only once. The paired samples *t* test showed a statistically significant improvement (practice effect) from the first to the second assessment for all tests given on two occasions except the Jumping and Clapping and One Board Balance Tests. Scores on the Stork Balance Test decreased with repeated assessment.

Motor Co-ordination (*r* = .512, n = 300, *p* < .01), Balance (*r* = .351, n = 300, *p* < .01), and the Overall Motor Index (*r* = .510, n = 300, *p* < .01) had moderate to strong correlations with Executive Function. All three motor composite scores had weak associations with Verbal Memory: Motor Co-ordination, *r* = .144, n = 300, *p* = .013; Balance, *r* = .176, n = 300, *p* = .002; Overall Motor Index, *r* = .189, n = 300, *p* = .001.

### Differences in performance according to background characteristics

#### *Constituent motor scores*

The distribution of scores obtained on the motor tests varied according to thebackground variables tested (Tables 3 and 4).

**Table 3:** **Distribution of gross motor test raw scores according to background characteristics, Mean (SD)**

Variable	N	Stork Balance	Ball Balance	Hopping in Squares	Jumping and Clapping	One Board Balance
Gender						
Boys	148	6.44 (3.27)	8.94 (2.14)	8.36 (3.57)	1.87 (.78)	2.33 (1.93)
Girls	160	6.82 (3.32)	9.38 (2.72)	9.42 (3.39)	1.74 (.74)	2.54 (2.14)
Age						
≤ 8 yrs	72	5.74 (3.39)	8.11 (3.01)	7.96 (3.58)	1.63 (.78)	2.07 (2.02)
8.5 - 9.0 yrs	108	6.32 (3.45)	9.24 (2.26)	8.56 (3.62)	1.73 (.72)	2.26 (2.00)
≥ 9.5 yrs	128	7.41 (2.95)	9.70 (2.09)	9.74 (3.21)	1.97 (.75)	2.80 (2.05)
Nutritional status						
Stunted	74	6.26 (3.42)	8.92 (2.94)	8.45 (3.91)	1.68 (.846)	2.65 (2.21)
Not stunted	234	6.76 (3.25)	9.25 (2.29)	9.06 (3.37)	1.85 (.725)	2.38 (1.98)
Household resources						
Level 1	123	6.59 (3.29)	9.12 (2.72)	8.73 (3.70)	1.74 (.76)	2.37 (2.10)
Level 2	94	5.97 (3.18)	9.09 (2.49)	8.98 (3.54)	1.81 (.82)	2.28 (1.93)
Level 3	91	7.38 (3.30)	9.32 (2.06)	9.09 (3.51)	1.89 (.69)	2.70 (2.07)
School exposure						
None	35	5.17 (3.47)	8.14 (3.63)	7.37 (4.35)	1.34 (.76)	1.43 (1.93)
1-2 years	101	6.65 (3.20)	8.97 (2.77)	8.30 (3.68)	1.81 (.81)	2.73 (2.09)
> 2 years	172	6.92 (3.26)	9.49 (1.85)	9.59 (3.05)	1.90 (.69)	2.48 (1.98)
Area of residence						
Rural	245	6.64 (3.24)	9.19 (2.60)	8.68 (3.69)	1.78 (.78)	2.42 (2.05)
Urban	63	6.60 (3.54)	9.10 (1.84)	9.81 (2.57)	1.90 (.64)	2.54 (2.02)

**Table 4:** **Mean differences in raw scores for timed motor tests, Mean (SD)**

Variable	N	Pegboard	Bead threading	Bolt board
Gender				
Boys	148	8.59 (1.65)	9.65 (1.70)	9.16 (2.35)
Girls	160	8.77 (1.57)	9.81 (1.71)	8.99 (2.63)
Age				
≤ 8 yrs	72	8.07 (1.06)	9.13 (1.42)	7.89 (2.10)
8.5 - 9.0 yrs	108	8.36 (1.52)	9.50 (1.66)	8.89 (2.18)
≥ 9.5 yrs	128	9.29 (1.74)	10.27 (1.73)	9.89 (2.67)
Nutritional status				
Stunted	74	8.41 (1.75)	9.68 (2.02)	8.93 (2.77)
Not stunted	234	8.77 (1.56)	9.75 (1.59)	9.12 (2.40)
Household resources				
Level 1	123	8.79 (1.71)	9.89 (1.72)	9.22 (2.87)
Level 2	94	8.41 (1.58)	9.59 (1.70)	8.72 (2.15)
Level 3	91	8.81 (1.48)	9.66 (1.68)	9.24 (2.26)
School exposure				
None	35	7.80 (1.85)	8.90 (2.11)	7.74 (2.67)
1-2 years	101	8.47 (1.47)	9.79 (1.56)	8.72 (2.59)
> 2 years	172	8.99 (1.56)	9.86 (1.66)	9.55 (2.27)
Area of residence				
Rural	245	8.66 (1.62)	9.72 (1.72)	8.98 (2.45)
Urban	63	8.78 (1.58)	9.79 (1.65)	9.43 (2.67)

##### Gender

Although girls performed better than boys on most of the measures of motor performance, significant differences were only recorded for the Hopping in Squares and Ball Balance Tests. Absolute effect sizes (Cohen’s *d*) on all the tests ranged from .07 to .31 (Table 5).

**Table 5:** **Associations of background characteristics with age-standardised motor co-ordination, balance and composite motor scores**

Variable	Gender						Nutritional status						Area of residence					
	Boys		Girls				Stunted		Not stunted				Rural		Peri-urban			
	(n = 148)		(n = 160)				(n = 74)		(n = 234)				(n = 245)		(n = 63)			
Balance	M	SD	M	SD	*t* ^a^	*d*	M	SD	M	SD	*t* ^b^	*d*	M	SD	M	SD	*t* ^c^	*d*
Stork balance	-.06	.99	.05	1.00	-.96	-.11	-.23	1.00	.07	.99	-2.25*	-.30	-.00	.99	.00	1.04	-.01	-
Ball balance	-.04	.72	.18	.80	-2.60*	.29	-.08	.79	.12	.76	-1.97	-.26	.09	.80	.01	.62	.83	-.14
Hopping in squares	-.15	1.00	.15	.94	-2.70**	-.31	-.22	1.07	.08	.94	-2.34*	-.30	-.06	1.02	.27	.74	-2.94**	-.38
One board balance	-.05	.95	.05	1.04	-.86	-.10	.01	1.05	-.00	.98	.05	-	-.02	1.01	.06	.97	-.53	-.08
Motor co-ordination																		
Pegboard	-.05	.98	.06	.94	-.99	-.12	-.31	.95	.11	.94	-3.35**	-.44	-.01	.96	.08	.98	-.65	-.09
Bead threading	-.03	.94	.04	.98	-.64	-.07	-.16	1.11	.06	.91	-1.58	-.22	-.01	.95	.05	1.01	-.43	-.06
Bolt board	.04	.90	-.05	1.00	.83	.10	-.20	1.12	.05	.89	-1.77	-.22	-.04	.95	.13	.94	-1.29	-.18
Jumping and clapping	.06	.94	-.09	.97	1.42	.16	-.27	1.06	.06	.90	-2.46*	-.34	-.06	.98	.14	.86	-1.61	-.22
Composite scores																		
Balance	-.08	.61	.11	.66	-2.53*	-.30	-.13	.70	.07	.61	-2.35*	-.31	.00	.65	.09	.58	-.92	-.13
Coordination	.01	.69	-.01	.71	.21	.03	-.24	.79	.07	.65	-3.37**	-.43	-.03	.71	.10	.67	-1.32	-.19
Overall index	-.03	.55	.05	.60	-1.27	-.14	-.18	.66	.07	.53	-3.37**	-.42	-.01	.58	.09	.54	-1.31	-.18

*p < .05, **p < .01, ***p < .001, df = 306.

^a^Jumping and clapping (df = 109).

^b^Jumping and clapping (df = 109), Bead threading (df = 106) and Bolt board (df = 103).

^c^Jumping and clapping (df = 107), Ball balance (df = 121) and Hopping in squares (df = 130).

##### Nutritional status

Analysis revealed significant differences for the Stork Balance, Hopping in Squares, Jumping and Clapping and Pegboard tests in relation to stunting (Table 5), with children with growth retardation performing worse than those without. Effect sizes for nutritional status were between -.30 and -.44.

##### Household resources

Children with more household resources (Level 3) had significantly higher scores on the Stork Balance Test than those in Levels 1 (most poor) and 2 (moderately poor). An effect size (partial eta squared) of .04 was recorded (Table 6). The pair-wise comparison of the most poor and moderately poor groups was non-significant.

**Table 6:** **Associations of background characteristics with age-standardised balance, motor co-ordination and composite motor scores**

Variable	Household resources								School exposure							
	Level 1		Level 2		Level 3				None		1-2 years		>2 years			
	(n = 123)		(n = 94)		(n = 91)				(n = 35)		(n = 101)		(n = 172)			
Balance	M	SD	M	SD	M	SD	*F*	^2^	M	SD	M	SD	M	SD	*F*	^2^
Stork balance	-.06	.99	-.19	.95	.29	1.00	6.04**	.04	-.55	1.03	.05	.97	.08	.98	6.26**	.04
Ball balance	.02	.79	.08	.75	.15	.76	.743	.01	-.24	.83	.07	.82	.14	.72	3.52*	.02
Hopping in squares	-.09	1.03	.04	1.00	.11	.89	1.206	.01	-.52	1.18	-.13	1.03	.19	.85	9.58***	.06
One board balance	-.08	1.00	-.07	.96	.18	1.02	2.159	.01	-.60	.93	.19	1.01	.01	.96	8.42***	.05
Motor co-ordination																
Pegboard	.00	1.01	-.15	.92	.17	.92	2.54	.02	-.65	.95	-.06	.90	.18	.94	12.06***	.07
Bead threading	.03	1.00	-.05	.92	.03	.95	.221	.00	-.59	1.07	.10	.84	.07	.97	7.99***	.05
Bolt board	-.03	1.07	-.13	.85	.15	.88	1.94	.01	-.66	1.07	-.10	.91	.18	.89	12.81***	.08
Jumping and clapping	-.14	.93	-.01	1.02	.14	.89	2.42	.02	-.72	.87	.01	.98	.11	.90	11.89***	.07
Composite scores																
Balance	-.06	.66	-.04	.63	.18	.59	4.25*	.03	-.48	.69	.05	.66	.11	.58	13.03***	.08
Coordination	-.04	.74	-.08	.69	.12	.65	2.24	.01	-.65	.73	-.01	.68	.13	.63	20.88***	.12
Overall index	-.05	.61	-.06	.57	.15	.51	4.16*	.03	-.57	.63	.02	.56	.12	.50	23.67***	.13

*p < .05, **p < .01, ***p < .001.

df = 2,305.

##### School exposure

Children with more than two years of schooling had significantly higher scores than those with fewer years on all of the motor measures. Effect sizes (partial eta squared) on all these differences ranged from .02 to .08 (Table 6).

##### Area of residence

Children living in peri-urban areas had significantly higher scores than those living in rural areas on the Hopping in Squares Test (Table 5), with an effect size of -.38.

#### *Composite scores*

##### Static and dynamic balance

Gender, nutritional status, household resources and school exposure created significant differences in the composite score for Static and Dynamic Balance (Tables 5 and 6).

##### Motor coordination

Nutritional status and school exposure had significant effects on the Motor Coordination composite score (Tables 5 and 6).

##### Overall motor index

Significant differences due to nutritional status, household resources and school exposure were recorded on the Overall Motor Index. Details are presented in Tables 5 and 6.

#### *Multivariate findings*

We compared the unique contribution of individual variables to the models for the constituent and composite motor scores. Variance inflation factors were less than 2 for all motor outcomes indicating no substantial multicollinearity in all the models.

##### Constituent motor measures

While nutritional status, household resources and school exposure were associated with the Stork Balance Test scores in the univariate analysis, these effects ceased to be significant in the regression analysis. Gender alone was associated with the Ball Balance Test, *F*(3,303) = 4.337, *p* = .005. Together with nutritional status and school exposure, gender accounted for 11.6% of the variance observed on the Hopping in Squares Test, *F*(4,302) = 11.005, *p* < .001. Nutritional status and school exposure were the strongest predictors (*R*^2^ = .074) for the Jumping and Clapping Test scores, *F*(3,303) = 9.178, *p* < .001 (Table 7).

**Table 7:** **Regression analysis results for tests of static and dynamic balance**

Variable		Gender	Nutritional status	Household resources	School exposure
Stork Balance^a^	*B*	-	.209	.016	.066
	*SE B*	-	.136	.016	.037
	β	-	.090	.061	.111
Adjusted *R* ^2^ = .027	*t*	-	1.537	1.003	1.772
Ball Balance^b^	*B*	.240	.050	-	.049
	*SE B*	.087	.044	-	.027
	β	.156	.067	-	.106
Adjusted *R* ^2^ = .032	*t*	2.762**	1.148	-	1.808
Hopping in Squares^c^	*B*	.349	.145	-	.128
	*SE B*	.105	.053	-	.034
	β	.179	.154	-	.221
Adjusted *R* ^2^ = .116	*t*	3.317**	2.747**	-	3.778***

*p < .05, **p < .01, ***p < .001.

^a^ *F*(3,304) = 3.813, *p* = .010.

^b^ *F*(3,304) = 4.235, *p* = .006.

^c^ *F*(3,304) = 14.797, *p* < .001.

Nutritional status and school exposure were associated with the Pegboard Test scores. School exposure alone contributed to the variance in the Bead Threading and Bolt Board Test scores (Table 8).

**Table 8:** **Regression analysis results for tests of motor co-ordination**

Variable		Nutritional status	School exposure
Pegboard^a^	*B*	.160	.126
	*SE B*	.053	.032
	β	.172	.221
Adjusted *R* ^2^ = .093	*t*	3.049**	3.909***
Bead Threading^b^	*B*	.104	.089
	*SE B*	.054	.033
	β	.112	.156
Adjusted *R* ^2^ = .040	*t*	1.917	2.686**
Bolt Board^c^	*B*	.075	.148
	*SE B*	.052	.032
	β	.081	.262
Adjusted *R* ^2^ = .081	*t*	1.423	4.607***
Jumping and Clapping^d^	*B*	.162	.094
	*SE B*	.053	.035
	β	-176	.165
Adjusted *R* ^2^ = .074	*t*	3.070**	2.695**

*p < .05, **p < .01, ***p < .001.

^a^ *F*(2,304) = 16.775, *p* < .001.

^b^ *F*(2,304) = 7.394, *p* = .001.

^c^ *F*(2,304) = 14.482, *p* < .001.

^d^ *F*(2,305) = 13.156, *p* < .001.

##### Composite motor scores

The models for the composites of Motor Co-ordination, *F*(2,304) = 25.043, *p* < .001, Static and Dynamic Balance, *F*(4,302) = 7.070, *p* < .001, and the Overall Motor Index, *F*(3,303) = 15.295, *p* < .001, were significant. Nutritional status and school exposure were associated with the Motor Co-ordination Composite. Gender and school exposure were associated with the composite score for Static and Dynamic Balance. Gender and school exposure also accounted for significant variance in the Static and Dynamic Balance Composite score. Nutritional status and school exposure accounted for 12.3% of the variance observed on the Overall Motor Index scores (Table 9).

**Table 9:** **Regression analysis results for composite scores**

Variable		Gender	Nutritional status	Household resources	School exposure
Balance^a^	*B*	.211	.059	.007	.073
	*SE B*	.071	.035	.010	.023
	β	.165	.096	.042	.191
Adjusted *R* ^2^ = .074	*t*	2.978**	1.673	.695	3.103**
Coordination^b^	*B*	-	.126	-	.117
	*SE B*	-	.037	-	.023
	β	-	.186	-	.280
Adjusted *R* ^2^ = .136	*t*	-	3.361**	-	5.078***
Overall motor index^c^	*B*	-	.094	-.002	.097
	*SE B*	-	.031	.009	.020
	β	-	.169	-.013	.283
Adjusted *R* ^2^ = .123	*t*	-	3.043**	-.224	4.734***

*p < .05, **p < .01, ***p < .001.

^a^ *F*(4,303) = 7.078, *p* < .001.

^b^ *F*(3,304) = 17.227, *p* = .001.

^c^ *F*(3,304) = 15.755, *p* < .001.

## Discussion

The current study documents the performance of school-age children on static and dynamic balance, as well as motor co-ordination tests. The stimulus materials used were simple to develop, not time-consuming and children participated willingly, demonstrating their suitability. Furthermore, the tests were inexpensive to develop and could be easily administered by trained testers. The developed motor measures were culturally appropriate and psychometrically sound with moderate to excellent reliability levels. Moderate to strong correlations of the motor scores with executive function scores provided evidence of convergent validity; on the other hand, weak associations with verbal memory demonstrated evidence of discriminant validity. Consistent with Bronfenbrenner’s bioecological model (Bronfenbrenner and Ceci 1994), we were able to identify proximal and distal influences on motor proficiency in school-age children.

### Influence of background characteristics

The superior performance of girls on the tests of dynamic balance is similar to what has been reported among South African (Portela 2007; du Toit and Pienaar 2002), Nigerian (Toriola and Igbokwe 1986) and Australian (Livesey et al. 2007) children. And congruent with the conclusions of Largo and colleagues (2003), gender differences on the various tasks varied in size and direction. Despite the differences observed in the current study, our findings do not however support the suggestion by Livesey and colleagues (2007) that separate gender-specific norms be used in the assessment of motor abilities in school-aged children. Reported differences between boys and girls within the studied age-group may have resulted from differences in cultural expectations – the socialising influences of parents and teachers – and environmental practices, as has been emphasized by others (Bénéfice et al. 1999; Thomas and French 1985; Munroe and Munroe 1975). In many rural communities such as the one in which the current study was conducted, girls are socialised to perform household activities from a young age. To successfully perform some of these tasks, such as fetching water from the river, requires balance.

Nutritional status was an important determinant of motor performance as it had moderate effects on balance and co-ordination. Children with growth retardation achieved lower scores on the composite motor test scores, similar to what has been reported in varied contexts from studies among younger (Bénéfice et al. 1999; Bénéfice et al. 1996; Abubakar et al. 2008b), older (Chang et al. 2010) and children of comparable ages (Chowdhury et al. 2010; Kar et al. 2008). The negative impact of poor nutritional status on motor performance may be attributed to deficiency in muscular strength (Malina and Little 1985), low energy levels (Dufour 1997) and slower motor development ((Malina 1984). Given that the negative impact of chronic undernutrition is long-term (Hoorweg and Stanfield 1976), and that stunting has a particularly strong effect on early gross motor development (Pollit et al. 1994), opportunities for interventions to specifically improve children’s nutritional status, should be explored.

Contrary to our expectations, children from the least wealthy households had lower scores than their counterparts from wealthier households only on the balance composite score. Furthermore, children from households with moderate wealth levels performed the worst on the Stork Balance Test and on the Overall Motor Index. The moderate effects sizes recorded suggested only modest differences among the various groups, demonstrating that socioeconomic conditions did not have such a major influence on children’s motor performance. These findings are in contrast to those reported in studies among populations with similar socioeconomic characteristics (Chowdhury et al. 2010). We offer the following explanations for our findings. As both nutritional status and household resources showed similar effect sizes in their associations with motor outcomes, it may be that the two are inextricably linked. For one, poorer households have fewer resources at their disposal and are therefore more likely to make poor nutrition-related choices. Second, our findings that nutritional status had a more pervasive role than SES may be related to the measure of stunting used. Height-for-age as a measure of chronic undernutrition may in itself be indicative of the cumulative effects of poor nutrition which impacts outcomes from a young age. Infant data from an earlier study in this area (Abubakar et al. 2008b) suggested that SES (conceptualised as distal factors) had less of an impact on child outcome than proximal factors (such as anthropometric status). Among our school-age population, we anticipated that SES would play a more influential role as the impact of outside environments surpasses that of immediate environments. The specific pathways through which poor SES and nutritional status affect outcome remain an area for further study.

Schooling effects were consistently larger than those of the other background influences suggesting that school exposure exerted a much stronger influence on child outcomes. Our findings have precedence in this setting where previous studies have reported strong consistent effects of school attendance on children’s performance (Alcock et al. 2008; Holding et al. 2004). Superior performance in children with greater exposure to school may, as has been postulated elsewhere (Bénéfice and Ba 1994), be attributed to the positive effects of attending school; the ability to follow instructions, pay attention to tasks and increased opportunities for practice.

With area of residence, the pattern of motor performance observed in the current study was unexpected as children living in the more rural areas had significantly lower scores on the Hopping in Squares Test. These findings were in stark contrast to reports from elsewhere which demonstrate that rural children consistently outperform their urban counterparts on tests of motor abilities (Portela 2007), since they have much more open play areas and they are more likely to engage in outdoor activities for longer periods of time (Loucaides et al. 2004). It should be noted that a much wider (and significant) variance in the mean scores of three tests for rural children in the current study possibly affected the significance levels recorded and may have jeopardized the validity of the obtained results (Glass et al. 1972). Perhaps we did not observe the expected differences in performance due to the widely disparate numbers of children in the two groups, reflecting a misclassification according to area of residence. Furthermore, our data failed to suggest that area of residence was a confounder on school attendance. Secondly, because we did not have a truly urban population, variations in the living conditions of children residing in rural and peri-urban areas may have been too subtle to create any real differences.

### Multivariate findings

After accounting for the effects of age, various predictors, created differences on the constituent motor scores, in isolation and collectively. Environmental (context) variables accounted for a greater proportion of the variance seen in test scores than biological (person) variables. These findings are in line with Bronfenbrenner’s (Bronfenbrenner 1999) model which stipulates that various aspects of the child’s environment have differential effects on development. Being male and having fewer years of schooling were risk factors for poorer scores on the balance composite scores, while growth retardation and less exposure to school were associated with poorer outcomes on the motor co-ordination composite and the Overall Motor Index. Compared with the other predictors, school exposure remained a consistent and strong influence on the composite scores.

## Conclusions

The current study provides preliminary evidence of motor performance from a typically developing rural population within an age range that has not been previously studied. As well as being culturally appropriate, the developed tests were reliable, valid and sensitive to biological and environmental correlates. Further, the use of composite scores seems to strengthen the magnitude of differences seen among groups. These correlates should be taken into account when assessing motor performance of school-age children living in similar contexts.

With strong ceiling effects, the Hopping in Squares Test which closely mimics a game that children within this context regularly engage in, seemed to be too easy. However, we recommend its inclusion in future batteries because it was sensitive to a number of the background influences tested. Imposing more stringent cut-offs for success will possibly increase the difficulty level of the test. On the other hand, we recommend the exclusion of the One Board Balance Test from test batteries because apart from strong floor effects, there were non-significant effects for all background influences apart from school exposure. In addition to small effect sizes, schooling effects disappeared when we included other predictors. The remaining tests performed well and their use in similar settings is recommended.

The children in the current study constituted a typically developing population at low risk for motor problems. The generally small to moderate effect sizes observed in the current study may be due to the types of comparisons being made or predictors considered. Larger effects may well be observed, for example, when comparing cognitive/motor skills in children with a neurological disorder (e.g. HIV or cerebral malaria) to those without a disorder. The sensitivity of 79% and specificity of 78% of the TQQ for detecting severe cognitive impairment suggests the need for a further screening procedure to detect those with mild or moderate cognitive impairment. Indeed, because we did not do further specific visual and audiological testing, impairments in these areas of functioning may have contributed to variability in performance on the more complex motor tasks. Further research with a more high-risk sample will provide an opportunity to test the clinical validity of the measures of motor performance.

## Abbreviations

CNS: Central Nervous System
ICC: Intraclass Correlation Coefficient
KEMRI/NERC: Kenya Medical Research Institute/National Ethics Review Committee
Movement-ABC: Movement – Assessment Battery for Children
SES: Socioeconomic status
TQQ: Ten Questions Questionnaire.
